# Can egg donor selection be improved? - a pilot study

**DOI:** 10.1186/1477-7827-8-76

**Published:** 2010-06-24

**Authors:** Norbert Gleicher, Andrea Weghofer, David H Barad

**Affiliations:** 1 Center for Human Reproduction (CHR) - New York and Foundation for Reproductive Medicine, NY, USA; 2 Department of Obstetrics, Gynecology and Reproductive Sciences, Yale University School of Medicine, New Haven, CT, USA; 3 Department of Obstetrics and Gynecology, University of Vienna Medical School, Vienna, Austria; 4 Departments of Epidemiology and Social Medicine as well as Obstetrics, Gynecology and Women's Health, Albert Einstein College of Medicine, Bronx, NY, USA

## Abstract

**Background:**

Accurate assessments of ovarian reserve (OR) in egg donor candidates are crucial for maximal donor selection. This study assesses whether recently reported new methods of OR assessment by age-specific (as-), rather than non-as (nas-) hormones, follicle stimulating hormone (FSH) and anti-Müllerian hormone (AMH), and triple nucleotide (CGG) repeats on the FMR1 (fragile X) gene have the potential of improving egg donor selection.

**Methods:**

Seventy-three consecutive egg donor candidates (candidates), amongst those 21 who reached egg retrieval (donors), were prospectively investigated for as-FSH, as-AMH and number of CGG repeats. Abnormal findings were assessed in candidates and donors and oocyte yields in the latter were statistically associated with abnormal FSH and AMH (>/< 95% CI of as-levels) and with normal/abnormal numbers of CGG repeats (normal range 26-32).

**Results:**

Amongst candidates mean as-AMH was 3.8 +/- 2.8 ng/mL (37.0% normal, 3.0 +/- 0.7 ng/mL; 26.6% low, 1.5 +/- 0.5 ng/mL; and 37.0% high, 5.8 +/- 2.2 ng/mL). AMH among donors was 4.2 +/- 1.7 ng/mL (33.3% normal, 14.3% low, and 52.4% high), yielding 17.8 +/- 7.2 oocytes, 42.9% in normal range (10-15), 9.5% in low (less than or equal to 9) and 47.6.% in high range (16-32). Candidates in 41.9% and donors in 38.1% demonstrated normal CGG counts; the remained were mostly heterozygous abnormal.

**Discussion:**

Prospective assessment of even carefully prescreened candidates and donors still demonstrates shortcomings on both ends of the OR spectrum. Utilization of ovarian reserve testing methods, like as-hormones and CGG repeats on the FMR1 gene have potential of improving candidate selections.

## Background

Oocyte donor selection in the United States (U.S.) represents a highly complex process, catering to different, guidelines and regulations [1]. A dominant medical need, not affected by federal regulations, is to ascertain normal ovarian function: Prematurely diminished ovarian reserve (PDOR) in young donor candidates will negatively affect oocytes yields [2], while excessive ovarian response to stimulation can not only result in poor oocytes quality [3] but lead to ovarian hyperstimulation syndrome (OHSS), and endanger the physical wellbeing of potential donors [4].

As for infertility patients, ovarian reserve (OR) in oocyte donor candidates is traditionallyassessed via baseline follicle stimulating hormone (FSH), especially at younger ages reported to be a rather poor tool to detect PDOR [2]. Anti-Müllerian hormone (AMH) appears to better reflect OR [5], and is, therefore, increasingly utilized to detect diminished ovarian reserve (DOR) [6,7] and/or hyperstimulation risk in women with polycyctic ovarian syndrome (PCOS) [8]. No studies on AMH utilization in oocyte donor selection have, however, so far been performed. This study does this.

Assessments of OR in infertile women as well as oocytes donors, historically, practically exclusively, involve non-age-specific (nas-) levels of FSH and/or AMH. FSH, however, increases, and AMH decreases with advancing female age, which makes such non-discriminatory use of normal cut off values appear illogical. We, therefore, proposed utilization of age-specific (as-) cut off values for FSH [2] and AMH [5], both found to be superior in determining OR: AMH at all ages [2,5] and FSH under age 38 years discriminate better between higher and poorer oocytes yields with in vitro fertilization (IVF) than previously utilized nas-values [2,6,9]. The utilization of as-AMH can also be helpful in determining ovarian hyperstimulation risk [5]. How as-OR testing impacts oocyte donor selection has, however, so far, not been investigated. This study investigates this issue.

Numbers of CGG triple nucleotide repeats on the *FMR1 *gene statistically relate to risk towards PDOR [10-12], with 26 to 34 repeats representing a normal range of counts in regards to the genes effects on ovarian function [13,14] (to be differentiated from normal and abnormal ranges of repeats in regards to neuro/psychiatric risks associated with the gene). Deviations from this normal range denote specific ovarian aging patterns, defined by varying rates of decline in OR [14]. In an infertile population, CGG counts also correlate to OR, as reflected by oocytes yields in IVF [11]. A specific heterozygous (normal/low) CGG repeat pattern appears to predispose towards a normal weight polycystic ovary phenotype [12].

The evaluation of triple CGG counts on the *FMR1*gene, thus, also offers potentially important clinical information about OR in young women. What such evaluations could contribute to egg donor screening has, however, not previously been investigated, and represents, therefore, together with as-FSH and as-AMH, the third new tool evaluated in this study for its potential to improve oocytes donor selection.

## Methods

### Patient population

Since July, 2007, the center evaluated 164 applicants as potential egg donors who, based on a detailed questionnaire, qualified for further investigation. Amongst those, 73 (ages 19 to 33 years, mean 24.2 +/- 3.6) reached, after two interviews, a first laboratory screening stage, thus becoming candidates for egg donation. At this stage they underwent vaginal ultrasonography, random AMH and genetic testing, including determination of number of triple nucleotide (CGG) repeats on the *FMR1*(fragile X) gene, for which patients signed an *FMR1*-specific informed consent.

### Laboratory assays

Since FSH, in contrast to AMH, requires timed blood draws [5], it was only evaluated in candidates who became donors by reaching ovarian stimulation.

FSH, estradiol and AMH were evaluated as previously described [5]. In short, FSH and estradiol were obtained on cycle days 2/3 and assessed utilizing a standard enzyme-linked immunoabsorbent assay (ELISA, AIA-60011, Tosho, Tokyo, Japan). Only results in assay range were considered for statistical evaluation. AMH was also evaluated by ELISA.[DSL-10-14400 active Müllerian Inhibiting Substance/Anti-Müllerian Hormone (MIS/AMH) enzyme-linked immunoabsorbent (ELISA), Diagnostic System Laboratories, Inc., Webster, TX 77598-4217, USA], an enzymatically amplified two-site immunoassay, which does not cross-react with other members of the TGF-β super family, including TGF-β1, BMP4 and ACT [15]. Theoretical sensitivity, or minimum detection limit, calculated by interpolation of mean plus two standard deviations of eight replicates of the 0 ng/mL MIS/AMH Standard, was 0.0006 ng/mL. Intra-assay coefficient of variation for an overall average AMH concentration was reported as ≤ 10 percent [15] and in our hands < 15%. Results are presented in ng/mL, with a conversion factor of 7.14 to pmol/L [16].

**Figure 1:**
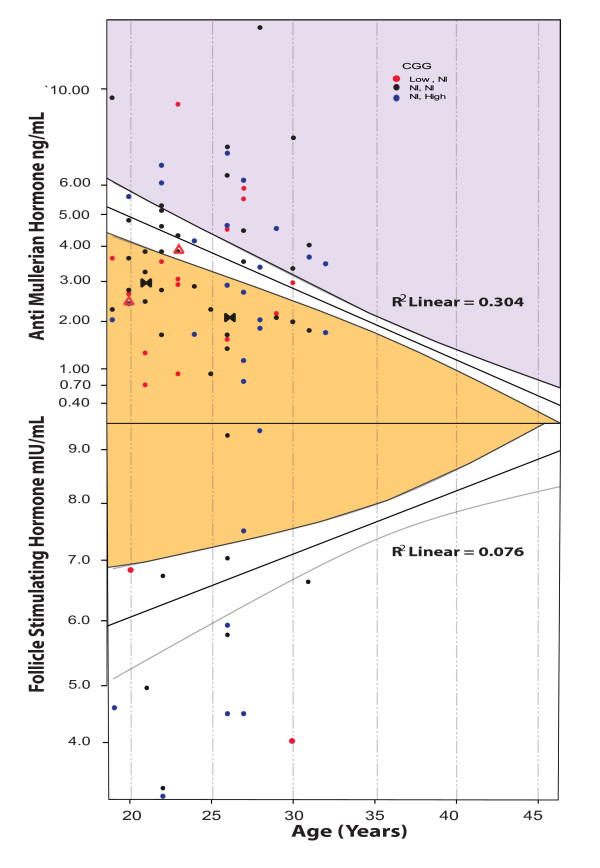
***as*-FSH and AMH levels**. *as*-FSH and *as*-AMH levels in candidates are shown against normal *as*-ranges previously established [5]. As the figure demonstrates, a considerable number of candidates demonstrate values outside of normal *as*-range for both, AMH and FSH.

Normal as-AMH and FSH levels have previously been published, based on 95%confidence intervals (CI) at all ages [2,5]. Figure 1 summarizes as-FSH and AMH levels as recently reported elsewhere [7].

Numbers of triple CGG repeats on the *FMR1 *gene were evaluated, utilizing commercial assays, as also previously described [10,11]. In short, *FMR1 *assays were performed testing DNA by Southern blot and polymerase chain reaction (PCR) to determine size and methylation status of CGG repeats. Southern blot analysis was performed using probe St.B12.3 on EcoR1 and Eagle digested DNA. PCR products were generated using fluorescent labeled primer, and were sized by capillary electrophoresis.

We previously defined and reconfirmed a normal range of CGG repeats as 26 to 34, with median at 30 repeats [13,14]. Women who's both allele counts fell into this range were considered normal; Those who demonstrated one allele in range and one outside, were considered heterozygous-abnormal, with normal/low (norm/low) and normal/high (norm/high) being separately evaluated. Those with both alleles in abnormal range were considered homozygous- abnormal.

### Statistics

All data are expressed as mean +/- standard deviation (SD); a p-value < 0.05 was considered statistically significant. Differences between normally distributed variables were tested with analysis of variance or covariance. Differences between groups of variables not conforming to normality were tested for with the Mann-Whitney test. All analyses were carried out with SPSS software for Windows version 17.0, 2005 (SPSS Inc. Chicago, IL.)

### Institutional Review Board

Our center's Institutional Review Board allows review of medical records for research purposes under expedited review, as long as patients sign an appropriate informed consent. This consent warrants that the chart review will maintain their anonymity and protect the patients' identity. These conditions were met for this study. In addition, all patients also signed a *FMR1*-specific consent.

## Results and Discussion

Amongst a total of 164 consecutive egg donor applicants, 73 reached initial AMH evaluations and *FMR1 *gene analyses and, thus, became donor candidates. As described in Table 1, their mean age was 24.2 +/- 3.6 years (range 19 - 34) and mean AMH was 3.7 +/- 2.8 ng/mL (range 0.6 to 13.0).

**Table 1:** Characteristics of donor candidates and donors

	Candidates	Donors
	(n = 73)	(n = 21)
Age (years; mean ± SD)	24.2 ± 3.6	24.1 ± 3.5
Total oocyte yield	n/a	17.8 ± 7.2
AMH (ng/mL; mean ± SD)	3.7 ± 2.8	4.2 ± 1.7
*as*-AMH normal (% of total^1^)	3.0 ± 0.7 (37.0)	3.2 ± 0.6 (33.3)
Oocyte yield^2^	n/a	20.7 ±9.1
low	1.5 ± 0.5 (26.0)	1.8 ± 0.2 (14.3)
Oocyte yield^2^	n/a	15.7 ± 3.8
high	5.8 ± 2.2 (37.0)	5.4 ± 1.4 (52.4)
Oocyte yield^2^	n/a	16.5 ± 6.4
FSH (mIU/mL: mean ± SD)	n/a	5.9 ± 2.0
*as*-FSH normal (% of total^1^)	n/a	5.0 ± 1.3 (76.5)
high	n/a	8.5 ± 1.3 (23.5)
*FMR1*: number of CGG repeats		
Allele-1	29.1 ± 3.3	29.0 ± 3.2
Allele-2	32.4 ± 2.2	32.2 ± 2.0
*Normal*^3 ^n (%)	30 (41.1)	9 (42.9)
AMH (ng/mL; mean ± SD)	3.3 ± 1.6	4.0 ± 1.5
*Heterozygous *3 n (%)	39 (53.4)	12 (57.1)
AMH (ng/mL; mean ± SD)	3.9 ± 2.7	4.0 ± 1.6
*Het-norm/low *n (%)	15 (20.6)	4 (19.0)
AMH (ng/mL; mean ± SD)	3.3 ± 2.3	3.9 ± 1.2
*Het-norm/high n *(%)	24 (32.9%)	8 (38.1)
AMH (ng/mL; mean ± SD)	4.4 ± 3.0	4.0 ± 1.9
*Homozygous*^3 ^n (%)	4 (5.5)	0
*Hom-high/high*	2 (2.7)	-
*Hom-low/high*	2 (2.7)	-
*Hom*-low/low	0	0

The Table summarizes patient characteristics in 73 consecutive egg donor candidates, who after an elimination process (for details see Materials and Methods section) reached a first laboratory testing stage and, from amongst them 21 who reached egg retrieval (one donor did not have *FMR1 *testing) (*donors*).

Candidates and donors did not differ statistically in any parameter.

^1 ^Reflects percentage of patients in total group.

^2 ^AMH does not distinguish between donors with normal or abnormal yields (p = 0.83).

^3 ^Normal is defined by both alleles demonstrating between 26-34 CGG repeats; in heterozygous women one allele demonstrates a count outside of this normal range, with *het-norm/low *defining an abnormally low and *het-norm/high *and abnormally high count.

Amongst those, 27 (37.0%) demonstrated AMH values within a normal as-range (mean, 3.0 +/- 0.7 ng/mL); 19 (26.0%) had low as-AMH, suspicious of DOR, (mean 1.5 +/- 0.5 ng/mL); and another 27 (37.0%) demonstrated abnormally elevated as-AMH levels, suspicious of PCOS (mean 5.8 +/- 2.2 ng/mL) and, therefore, potentially reflected hyperstimulation risk (Figure 1). Only 17 candidates had baseline FSH levels, and those ranged from 3.1 to 9.8 mIU/mL (Figure 1).

**Figure 2:**
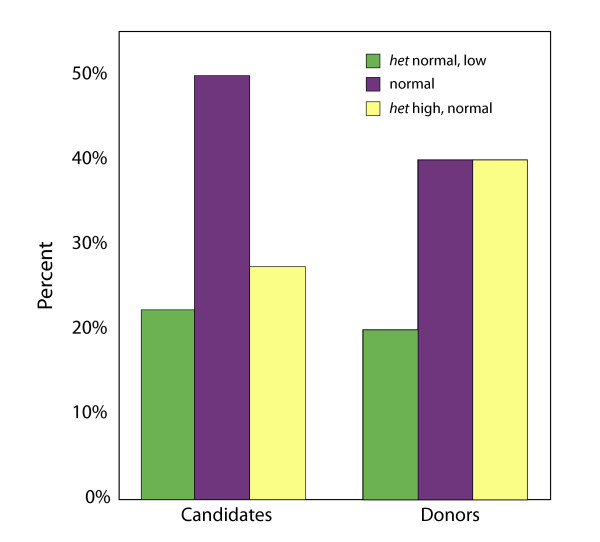
**Distribution of CGG counts on *FMR1 *gene in candidates**. The differences in distribution were not significant.

*FMR1 *analyses amongst candidates demonstrated normal distribution of CGG repeats on both alleles in 30 (41.1%), with 39 (53.4%) being heterozygous abnormal, amongst those 15 (20.6%) being het-norm/low and 24 (32.9%) being het-norm/high. Four candidates (5.5%) were homozygous, two (2.7%) each hom-low/high and hom-high/high but none were hom-low/low. (Table 1). Overall mean distribution of CGG repeats on both alleles did not vary significantly between candidates and donors (Table 1, Figure 2).

Analysis of oocytes yield in 21 candidates who by time of study analysis became donors and, therefore, reached oocytes retrievals allowed limited correlations to FSH, AMH and *FMR1 *status. Mean age was 24.1 +/- 3.5 years (range 19 - 34) and they yielded 17.8 +/- 7.2 oocytes (range 6 - 32). AMH levels were 4.2 +/- 1.7 ng/mL (range, 1.6 - 7.9) and FSH levels 5.9 +/- 2.0 mIU/mL (range 3.1 - 9.8) (Table 1).

A total of 14/21 (66.7%) of donors demonstrated abnormal as-AMH; amongst those 3/21 (14.3%) abnormally low and 11/21 (52.4%) abnormally high values. Values of as-FSH were abnormally elevated in 4/17 donors (23.5%) and normal in 13/17 (76.5%).

CGG counts were normal in 9/21 (42.9%) and heterozygous-abnormal in 11/21 (57.1%) of donors, 4/21 (19.0%) with heterozygous genotype of norm/low and 8/21 (38.1%) with norm/high (Table 1).

Investigating abnormalities in laboratory findings as predictors of oocytes yields, 2/21 (9.5%) donors produced abnormally low (≤ 9) oocytes numbers (7 and 6, respectively), 10/21 (47.6%) produced abnormally high oocytes yields (range 16-32) and only 9/21 (42.9%) showed egg numbers considered to be in a normal range of 10-15 (Table 2). In two donors with abnormally low oocytes production AMH was normal and high, respectively; FSH was available in only one and was normal; and both demonstrated heterozygous abnormal CGG counts, one normal/high and the other normal/low.

Subgroups were too small to reach statistically valid conclusions, cross-tabulating oocytes yields and as-AMH levels. Donors with abnormally high oocytes yields demonstrated in 40.0% normal as-AMH, in 50.0% abnormally high and in 10.0% abnormally low values. Donors with normal oocytes numbers demonstrated normal as- AMH in 22.2%, abnormally high levels in 55.6% and abnormally low as-AMH in 22.2%. AMH statistically did not distinguish between normal and abnormally high oocytes yields (p= 0.83).

Six out of seven donors (85.7%) with high oocytes yields demonstrated normal as-FSH, while only 4/7 (57.1%) with normal egg numbers showed normal FSH values and 3/7 (42.9%) elevated as-FSH. These differences were, however not statistically different.

Investigating associations between oocytes yields and CGG counts, all nine women with counts on both alleles in normal range (26-34) demonstrated either normal (6/9, 66.6%) or high (3/9, 33.3%) egg numbers. None had abnormally low oocytes yields. Otherwise, small patient numbers did not allow for further statistically robust conclusions.

In the U.S., the selection of oocytes donors has become increasingly complex since the Food and Drug Administration (FDA) has assumed regulatory authority [1]. The FDA's interests primarily, however, extend to infectious transmission risks. Other aspects of candidate selection are left to patients and physicians. Oocytes (and embryo) yields are, amongst those, of great importance. Correct assessments of OR in oocyte donation candidates represent, therefore, a very essential part of the egg donation process.

A number of perceptive papers recently well summarized progress in assessing OR [17-20]. Most concentrated on the utilization of AMH, with consensus evolving that AMH may, overall, be a better reflection of OR than baseline FSH [5,8,9,21]. This should not surprise: While AMH and FSH correlate [22], both, in principle, reflect different stages of follicular maturation, AMH small, preantral and FSH more mature, preovulatory follicles [17]. As OR is defined to reflect remaining follicles in ovaries, the larger preantral pool should, indeed, better reflect OR than preovulatory follicles, primarily represented by FSH.

Though predictive values of FSH [2,23] and AMH [5] change as females age, and even though as-evaluations offer distinct advantages over nas-testing [2,5], OR evaluations still, almost uniformly, utilize nas-normal ranges, independent age. The former, however, also can be expected to improve OR assessments in oocyte donation candidates. This study supports this assumption by demonstrating that a surprisingly large number of them fall outside normal as-OR testing parameters and are, therefore, at risk for either too low or to high oocytes yields. As Table 1 demonstrates, only 37.0 percent of candidates and 33.3 percent of donors demonstrated normal as-AMH, only 76.5 percent of donors normal FSH and only 41.1 percent of candidates and 42.9 percent of donors normal CGG counts.

as- FSH, as-AMH and CGG counts are new tools in defining OR. They never before were applied to selection of oocytes donation candidates. Since oocytes donation is a voluntary and costly process, risks to donors and costs to recipients have to be minimized. Improvements in OR assessments facilitate both: better risk prediction of excessive oocytes yields should eliminate most OHSS risks. Carlesen and associates reported that abnormally high as-AMH denote such risk [8]. At the other extreme, avoidance of disappointing oocytes yields, reduces unnecessary costs.

The frequency of as-AMH abnormalities was surprising. So far, only few studies reported cut-off values suggestive of DOR (and/or poor oocytes/embryo quality) [5,16] and of OHSS risk [8,9]. This study utilized as-AMH levels, based on 95% CIs of patients of all ages at our cente. Women with AMH below the lower cut off were considered at risk for DOR; those above the 95% CI for their age were considered at OHSS risk. These levels have previously been demonstrated to discriminate abnormally low and abnormally high oocytes yields [5].

Remarkably, only approximately one third of donation candidates (37.0%) demonstrated normal as-AMH; approximately a quarter (26.0%) exhibited abnormally low AMH and were, therefore, at risk for low oocytes production; and another remarkable 37.0 percent produced abnormally high levels, placing them at risk for OHSS.

Relatively small donor numbers prevent this pilot study from outright statistical conclusions on specific benefits from here utilized, new OR assessment techniques in oocytes donation cycles. Statistical trends, however, uniformly, and without exception, support a positive effect on donor selection: As Table 1 demonstrates, AMH levels increased from candidates to donors from 3.7 +/- 2.8 to 4.2 +/- 1.7 ng/mL. Moreover, AMH levels increased in candidates with normal as-AMH (3.0 +/-0.7 to 3.2 +/- 0.6 ng/mL) and low as-AMH (1.5 +/- 0.5 to 1.8 +/- 0.2 ng/mL) but decreased in candidates with high as-AMH who became donors (5.8 +/- 2.2 to 5.4 +/- 1.4 ng.mL).

These opposing trends, are, of course, exactly what one would expect as a consequence of better donor selection, with OR improving (improving AMH levels) from candidate status to donor status in women with lower OR and declining (declining AMH) from candidate to donor status in those with excessively high OR.

Though, especially amongst donors, subgroups became too small to reach statistically robust conclusions, correlations with oocytes yields, nevertheless, were remarkable (Table 2): 9.5 percent demonstrated low oocytes yields (≤ 9), approximately half, high oocytes numbers (16-32) and approximately 40% normal oocytes yield (10-15). Oocytes yields with normal (20.7 +/- 9.1 oocytes) and high as-AMH (16.5 +/- 6.4 oocytes) were higher than with low as-AMH (15.7 +/- 3.8 oocytes), though differences failed to reach significance. The data, however, correlate well with earlier reports that as-AMH discriminates between lower and higher oocytes yields [5,7].

**Table 2:** Percentage distribution of oocyte yields in donors

Oocyte yield	Normal	Low	High
(n oocytes)	10-15	(≥ 9)	(16-32)
n donors (%)	9 (42.9)	2 (9.5)	10 (47.6)
*as*-AMH normal (%)	28.6	14.3	57.1
low (%)	66.7	0	33.3
high (%)	45.5	9.1	45.5
*as*-FSH normal (%)^1^	57.1	100.0	85.7
high (%)	42.9	0	14.3
*FMRI *numbers of CGG repeats			
*Normal *(%)	66.6	0	33.3
*Heterozygous*^2^	-	-	-
*Homozygous*^2^	-	-	-

^1 ^FSH values did not statistically discriminate between normal and abnormal oocytes yields.

^2 ^Numbers too small for statistical evaluation;

The study, therefore, suggests that as-AMH may, indeed, improve egg donor selection. The large number of candidates, by here utilized criteria defined at risk for OHSS,would, however, suggest that as- 95% CIs may represent too low a cut off. Nelson et al defined OHSS risk even lower at nas-AMH above 15 pmol/L (2.1 ng/ml) [9]. A better definition of OHSS risk, therefore, awaits additional studies, preferably utilizing as-AMH values.

Candidates also demonstrated a significant prevalence of abnormal CGG counts on their *FMR1 *genes. This should, however, not surprise since Fu et al reported in normal populations a large distribution peak between 29 and 30 repeats but significant additional distribution at lower and higher numbes [24]. We found this peak intriguing and hypothesized that it may represent a normal distribution range in regards to potential OR-related function of the gene [13,14].

In this study a little less than half of all candidates (41.1%) and donors (42.9%) showed normal distribution, 53.4 percent of candidates and 57.1 percent of donors demonstrated heterozygous abnormalities. Only candidates (5.5%), but no donors, demonstrated homozygous abnormalities. Donors reaching retrieval, thus, demonstrated a very similar distribution pattern to candidates (Table 1).

Like general populations, egg donors can, therefore be expected to demonstrate significant CGG count abnormalities. Since such abnormalities may denote different time patterns in decline of OR, such information may be helpful in egg donor selection, especially if donor candidates are in their later 20ies, when *FMR1 *CGG count patterns can already significantly affect OR [14].

Here reported oocytes yield data have to be viewed with caution since, like in any retroactive analysis, they already reflect integration of knowledge from earlier screening stages. Some here reported donors, therefore, may have yielded fewer oocytes had they not been recognized as at risk towards DOR, and received appropriately modified stimulation. Integration of earlier OR screening data may, therefore, have possibly blunted otherwise more prominent differences. Similarly, IVF programs integrate clinical suspicion for PCOS into cycle management, whether based on the sonographic appearance of ovaries or high AMH levels.

## Conclusions

In summary, here presented data suggest that as-FSH, as-AMH and *FMR1 *testing may be helpful in further improving egg donor selection. To what degree, will, however, remain to be determined. Such a conclusion is also supported by general advantages of as-testing over nas-OR testing and very predictive patterns of ovarian aging associated with CGG repeats on the *FMR1 *gene.

## List of abbreviations

AMH: Anti-Müllerian hormone; as-: Age-specific; CI: Confidence interval; DOR: Diminished ovarian reserve; ELISA: Enzyme linked immunoabsorbent assay; FDA: Food and Drug Administration; *FMR1*: Fragile X mental retardation 1; FSH: Follicle stimulating hormone; het: Heterozygous; hom: Homozygous; IVF: In vitro fertilization; nas: Non-age-specific; norm: Normal; OHSS: Ovarian hyperstimulation syndrome; OR: Ovarian reserve; PDOR: Prematurely diminished ovarian reserve; PCOS: Polycystic ovarian syndrome; PCR: Polymerase chain reaction; SD: Standard deviation; US: United States

## Competing interests

NG and DHB, besides other parties, are co-inventors and co-owners of a U.S. patent, claiming therapeutic benefits from DHEA supplementation in women with diminished ovarian reserve. Additional DHEA patent claims are currently still pending. Both of these authors are also listed as co-inventors and co-owners of a pending patent application, claiming ovarian regulatory functions for the *FMR1 *gene. NG, AW and DHB have been recipients of research support, speaking honoraria and travel funds from various pharmaceutical and medical device companies, none related to the topic of this publication.

## Authors' contributions

NG and DHB contributed equally to this manuscript. Both participated in design of study and data analysis. NG drafted most of the manuscript, while DHB performed a majority of the statistical analysis. AW participated in design of study and reviewed the manuscript. All authors read and approved the final manuscript.
